# Does the ‘Mountain Pasture Product’ Claim Affect Local Cheese Acceptability?

**DOI:** 10.3390/foods10030682

**Published:** 2021-03-23

**Authors:** Isabella Endrizzi, Danny Cliceri, Leonardo Menghi, Eugenio Aprea, Flavia Gasperi

**Affiliations:** 1 Research and Innovation Centre, Department of Food Quality and Nutrition, Fondazione Edmund Mach, via E. Mach 1, 38010 San Michele all’Adige, Italy; leonardo.menghi@fmach.it (L.M.); eugenio.aprea@fmach.it (E.A.); flavia.gasperi@fmach.it (F.G.); 2 Center Agriculture Food Environment, University of Trento/Fondazione Edmund Mach, via E. Mach 1, 38010 San Michele all’Adige, Italy; danny.cliceri@gmail.com; 3 Department of Technology and Innovation, Center University of Southern Denmark, Campusvej 55, 5230 Odense, Denmark

**Keywords:** mountain cheese, acceptability, conjoint analysis, external information, consumer segmentation, food sustainability

## Abstract

This paper aims to explore the impact of “mountain pasture product” information on the acceptability of local protected designation of origin (PDO) cheese produced from the raw milk of cows grazing in mountain pastures (P) or reared in valley floor stalls (S). A total of 156 consumers (55% males, mean age 41 years) were asked to evaluate their overall liking on a 9-point hedonic scale of four samples: Cheeses P and S were presented twice with different information about the origin of the milk (cows grazing on mountain pasture or reared in a valley floor stall). Demographics, consumer habits, and opinions on mountain pasture practice (MPP), attitudes towards sustainability, and food-related behaviours (i.e., diet, food waste production, organic food, and zero food miles products purchase) were recorded and used to segment consumers. The cheeses were all considered more than acceptable, even though they were found to be significantly different in colour and texture by instrumental analyses. In the whole consumer panel, the cheese P was preferred, while in consumer segments less attentive to product characteristics, this effect was not significant. External information had a strong effect: Overall liking was significantly higher in cheeses presented as “mountain pasture product”, both in the whole panel and in consumer segments with different attitudes (except for those with a low opinion of MPP).

## 1. Introduction

Mountain dairies—which, in the alpine territories, are placed in contexts with a high naturalistic value, in most cases—play key roles in the promotion of local tourism, the preservation of biodiversity and the environment, and the maintenance of cultural and historical traditions [1]. They also find themselves in a position of increasingly seeking a compromise between production and conservation needs, as well as trying to respond convincingly to consumer requests regarding food safety and compliance with ethical farming principles. While animal husbandry, in general, has been subjected to criticism due to the excessive intensification and poor efficiency of production processes, mountain activity has gained growing interest from both tourists and consumers who associate this activity with greater sustainability, being able to combine production, environmental, and social needs on a small scale [2]. Nevertheless, mountain farming is less competitive and has higher costs than intensive production, with the consequent abandonment of such activities in the most remote areas in recent decades [3,4]. The EU has recognized, for some years, the need to prevent the abandonment of these mountain areas by focusing on the promotion and development of mountain food production as a way to promote sustainable development and to reduce the disadvantages of these areas [5]. With this objective, the EU has recently introduced a new labelling system for mountain products [6], which is an important step toward taking into account that there is a strong correlation between the perception of EU quality signs and the attitude towards food origin [7].

### 1.1. Mountain Products and Consumer Perception

The quality of dairy mountain products starts from the animals, which are moved from indoor feeding with conserved forage to fresh herbage (i.e., feeding on pasture), according to the traditional transhumance system [8]. Several studies have focused on the effects of summer grazing on dairy product characteristics, demonstrating that mountain products are different from indoor feeding products, in terms of sensory attributes [9,10,11,12,13], volatile organic compounds (VOCs)’ profile [8,14,15,16], and fatty acid composition [11,17,18]. The quality of dairy mountain products is also a key aspect for consumers [19], for whom the production of high-quality dairy products is the most important ecosystem service among those provided by summer farms [20].

### 1.2. Influence of Information and Attitudes

When the quality of a food product is evaluated, we have to take into account that consumer perception is based both on intrinsic characteristics (mainly related to product physical properties) and extrinsic properties (i.e., any information provided to the consumer about the product) [21]. Among the external factors, those linked to product credibility, such as labels, brands, origin, organic and production method-related, health, and ethics, are those of high interest for product enhancement in the market [22].

For food products of animal origin, some studies have investigated the effect of information about the production method, demonstrating that information about cow grazing versus information about indoor system increased consumer preference for meat [23,24] and generated the highest willingness to pay or positive effect, in terms of liking milk [25,26,27,28]. Romanzin et al. [28] have reported similar results for cheese, even if the literature on this food matrix is scarce. A recent review on consumer perception, preferences, and behaviours regarding pasture products did not report any study on cheese [29].

External factors generate expectations about food products and influence the choices of consumers, having a role in both their perception and liking [30,31]. In spite of this, the magnitude of these effects on food choice depends on how well the consumer is informed, aware, and prepared towards the concepts associated with the external information transmitted [32,33,34]. Studies have demonstrated that consumers associate sustainability-related attributes, such as environmental friendliness, animal welfare, local, and small-scale production, with mountain pasture products [2,35]. Nevertheless, it is important to measure consumer engagement with respect to the mentioned aspects, in terms of knowledge, awareness, and attitude. Some examples in the literature have proposed self-assessed measures or questionnaires for estimating the individual environmental sustainability [36], the low-carbon consumption scale [37], and knowledge of food sustainability [38]. Poortinga and Darnton [39] developed a screening tool to segment consumers, according to their attitude towards environmental, economic, and social aspects of sustainability. The developed tool has been proven to be valid in differentiating the Welsh population, even though it lacks a domain that measures the aspect linked to food sustainability.

### 1.3. Objective of the Study

In this study, we explored the impact of “mountain pasture product” information on the acceptability of a local cheese, with the final aim to enhance the value of dairy products obtained from milk produced in mountain pastures, thus promoting the multiple positive externalities connected to them. In order to identify the profiles of consumers differently involved with the mountain pasture world and differently inclined towards environmental and food sustainability, we developed and proposed new questionnaires as screening tools for this precise purpose. Then, we verified whether consumer segments with different attitudes toward these quality characteristics experienced a different impact of external information about mountain pasture products.

## 2. Materials and Methods

### 2.1. Cheese Samples

A protected designation of origin (PDO) cheese (Puzzone di Moena) was the product chosen for the consumer test. Puzzone di Moena is a semi-hard cheese with characteristic washed rind, produced from raw bovine milk of animals reared in the mountain area of Trentino Alto Adige (Italy) from a minimum height of 1000 m (valley floor stalls) up to 2000 m of altitude (mountain pasture). Puzzone di Moena is generally sold at 100 days of maturation, but the ripening period varies from a minimum of 3 up to a maximum of 16 months. Puzzone di Moena “malga cheese” is a Slow Food presidium [40], sold with the label “Sapori di malga” (which means “Mountain hut flavours”). It is exclusively produced from alpine pasture milk during the summer pasturing period, from June to September.

The samples given to the consumers were obtained from two different wheels of Puzzone di Moena PDO produced in Predazzo dairy (Trento, Italy): One made from milk collected in valley floor stalls (S) and aged 100 days, and the other one from alpine pasture milk (P) and aged 200 days. The two cheeses were considered representative of the two types of Puzzone present in the local market: The mountain pasture product is sold with greater maturation, in order to enhance its distinctive characteristics (min 120 days) [41], while the valley bottom cheese is mostly sold with a minor seasoning (min 60 days) [42].

#### Sample Preparation

Cheese wheels were stored at 15 °C until the moment of portioning, which took place the day before the test. To obtain homogeneous samples, the whole wheel was first cut in half, then into two quarters. From each quarter, eight 1.5-cm thick slices were cut. From each slice, after removing the rind, a parallelepiped was obtained, which was further divided into 16 smaller parts (3 × 1.5 cm × 1.5 cm). The cheese pieces were then stored in vacuum-sealed containers at 10 °C until the next day. On the day of the test, each piece of cheese was placed in a transparent bio-plastic cup, covered with a lid, coded with a three-digit number, and stored at 15 °C until tasting.

### 2.2. Physical and Rheological Properties

For each cheese (P and S), 32 cheese parallelepipeds randomly selected from those cut were collected (one piece for each cheese slice) and submitted first to instrumental measurements of colour and then of texture characteristics. Colour measurements were recorded at room temperature on freshly cut cheese slices using a tri-stimulus CR-400 colorimeter supported by the CM-S100wSpectraMagicTM colour data software (Konica Minolta Sensing, Inc., Tokyo, Japan) and calibrated with a white standard plate. The L*-, a*-, and b*-parameters of the CIEL*a*b* colour space model (see [43]) describe visual lightness (as values increase from 0 to 100), redness to greenness (positive to negative values, respectively), and yellowness to blueness (positive to negative values, respectively) of the samples.

Texture properties were then measured using a TA-XT texture analyser, equipped with an acoustic envelope detector device (Stable MicroSystem Ltd., Godalming, UK). A 4-mm probe was used to compress the samples. Nine mechanical parameters were calculated from the recorded curves, following the method described by Costa et al. [44].

### 2.3. Consumer Study

The consumer test was conducted in the Trento Expo exhibition spaces (Trento, Italy), on the 16 and 17 March 2019, in “La Casolara 2019”, the traditional Slow Food presidium fair dedicated to the best cheese and dairy production from all over the country, attracting not only local visitors.

The responses of 156 consumers were collected in a mobile sensory laboratory compliant with EN ISO standards 8589 [45], equipped with four mobile individual booths using the FIZZ 2.46A software (Biosystemes, Couternon, France).

**Figure 1:**
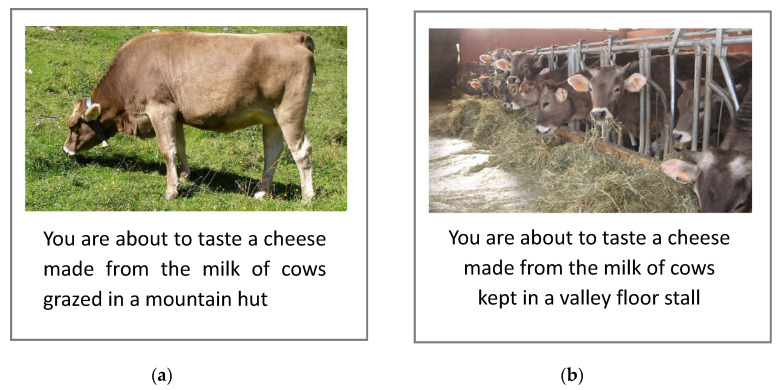
Examples of the screen used in the conjoint study: (**a**) The information about mountain pasture cheese; and (**b**) the information about cheese made with milk from cows reared in valley floor stalls.

The test consisted of an experiment that was evaluated in ‘informed’ conditions, combining conjoint analysis with the tasting of the two Puzzone di Moena PDO cheeses (described in Section 2.1). Each consumer received four cheese samples in total, according to a complete factorial design with two milk productions and two information levels. The two cheeses (P and S) were presented twice, each time with different external information: ‘Produced from milk of cows reared on mountain pasture’ (Claim_P) or ‘Produced from milk of cows reared in valley floor stalls’ (Claim_S). These two claims were submitted to consumers on the computer screen (Figure 1), just before tasting the sample. Consumers rated their overall liking of the four cheeses on a nine-point scale, from 1 = “Dislike extremely” to 9 = “Like extremely”. The four samples were presented in a random and balanced order for each participant, who evaluated them under white light.

All subjects were not paid and voluntarily joined the test. Prior to participation, the experimental procedure was explained to all participants and written informed consent was obtained from each, according to the European Data Protection Regulation (UE 679/2016). The consumers were asked to pay attention and to carefully read all the instructions provided during the test. We provided participants with noise-proof earmuffs, in order to help them concentrate in the noisy fair environment, and asked them to follow a rinse procedure with water and unsalted crackers to avoid possible carry-over effects between the products tested.

### 2.4. Questionnaires

After tasting, by means of a series of questionnaires, participants provided information about a list of different topics, from socio-demographic data to self-reported behaviours and habits related to food, sustainability, and mountain pasture perception (Table 1). They were asked about their food diet, in order to identify the omnivore, flexitarian (people reducing or limiting their meat consumption), and vegetarian/vegan distribution in the panel, using 9 items adapted from De Backer and Hudders [46], which have already been used in Italian [47,48]. Consumers reported their percentage of weekly food waste, as well as organic and zero food mile products weekly purchased (<5%, 5–10%, 11–20%, 21–30%, 31–40%, >40%). To assess interest towards natural products, the Natural Product Interest (NPI) sub-scale of the Health and Taste Attitude Scales (HTAS), developed by Roininen et al. [49] and validated by Saba et al. [50], was used. Participants rated their degree of agreement with a series of positive and negative statements on a 9-point scale (1 = totally disagree; 9 = totally agree), rather than the original 7-point scale, in order to be consistent with the other questionnaires submitted to the participants. In the Supplementary Materials, the original and the Italian version of the NPI sub-scale is reported (Table S1).

#### 2.4.1. Attitude towards Sustainability and Food Sustainability

Furthermore, participants provided information regarding their attitude towards sustainability (ATS), rating their degree of importance, agreement, and concern on the response option expected by each statement of the 15-item Welsh screening tool for sustainability [39]. The original scale was back-translated in Italian by a native bilingual, following the procedure suggested by Brislin [51]. In this case, a 9-point scale (1 = not important at all/totally disagree/not concerned at all; 9 = extremely important/totally agree/extremely concerned, depending on the statement), rather than the original 6-point scale with the escape answer “don’t know”, was used. In the original Welsh questionnaire, there were no statements investigating sustainability, in terms of food consumption. Given the importance for a study like this to collect this information, a list of 18 positive and negative statements investigating attitudes towards local food, green restaurants, and domestic food waste were developed, in order to cover the food consumption sustainability domain (FCS; Table 2). In the Supplementary Materials, the original Welsh questionnaire, its translation in Italian (ATS), and the Italian version of FCS scale are reported (Tables S2 and S3).

#### 2.4.2. Mountain Pasture Practice Values

Six further statements on the values and habits of mountain pasture practice (MPP) were developed, in order to explore the knowledge level and perception of the mountain pasture world and its products of consumers (Table 3). These statements were previously developed by a focus group of researchers involved in different fields related to mountain pasture and food (i.e., sensory, nutrition, chemistry, food technologies, animal husbandry, agricultural economics, and statistics). In the Supplementary Materials, the Italian version of the MPP scale is reported (Table S4).

### 2.5. Data Analysis

Data analysis was performed using the STATISTICA v. 13.1 software (Dell Inc., Tulsa, OK, USA, 2016).

In order to confirm the differences between the two cheeses (P and S), the product effect (fixed factor) on colour and texture instrumental parameters was estimated using one-way analysis of variance (ANOVA). In all statistical tests, we consider a significant difference as *p* < 0.05 after Bonferroni correction.

#### 2.5.1. Analysis of Questionnaire Data

As a preliminary pre-treatment, the scores for the negative statements of NPI questionnaire [49], of the ATS scale [39], and of the developed scales on FCS and statements on MPP were reversed. For the NPI scale, the statements suggested by the authors were considered negative while, for the ATS scale, we considered four statements (8–11) of the original scale negative, as they were found to be opposite to the others in an explorative principal component analysis (PCA) map (data not shown). Following the same procedure, of the 18 statements developed for FCS, eight were considered negative (Table 2); while the MPP statements were all considered positive, being true statements extracted from the literature.

Subsequently, the internal validity for each scale was tested using the standardized Cronbach’s alpha [58]. Cronbach’s alpha values above 0.60 are considered acceptable and values above 0.70 are considered good to optimal [59]. Then, for each of the four scales, the sum scores were calculated for each participant by adding the score of each item, according to the procedure described by Roininen et al. [49]. Based on these scores, the participants were classified in three groups (low, moderate, and high interest/attitude), according to the 33rd and 66th percentiles.

A two-way analysis of variance (ANOVA) was carried out to test how gender and age (three age classes were considered: Age_1, 18–30; Age_2, 31–50; and Age_3, 51–75) affected the sum scores of the attitude scales. One-way ANOVA was instead used to estimate the effect of food diet (omnivorous, flexitarian, or vegetarian/vegan), percentage of weekly food waste, organic, and zero food mile products weekly purchased (<5%, 5–10%, 11–20%, 21–30%, 31–40%, >40%) on attitude scale sum scores. For significant effects after Bonferroni correction (corrected *p* < 0.05), the Tukey–Kramer post-hoc honestly significant difference (HSD) test for unequal sample size was applied, whenever appropriate.

#### 2.5.2. Analysis of Conjoint Data

The liking data were analysed using a three-way ANOVA mixed model, considering both product and external information as main fixed factors and consumer as the random main factor, together with their second-order interactions. For significant effects after Bonferroni correction (corrected *p* < 0.05), the post-hoc HSD Tukey’s test for multiple comparison was applied, whenever appropriate.

In order to identify which groups of people were more sensitive to intrinsic or extrinsic factors, the same ANOVA model was recalculated in sub-groups of consumers, identified by gender, age (Age_1, 18–30; Age_2, 31–50; Age_3, 51–75 years of age), place of residence altitude (Alt_1, >600; Alt_2, 600–300; Alt_3, <300 m a.s.l.; [60]), residence zone (Urb_1, >150 inhab/km^2^; Urb_2, <150 inhab/km^2^; [61]), interest towards natural products (NPI_1, low; NPI_2, moderate; NPI_3, high), attitude towards sustainability (ATS_1, low; ATS_2, moderate; ATS_3, high), attitude towards food consumption sustainability (FCS_1, low; FCS_2, moderate; FCS_3, high), and attitude towards mountain pasture practice values (MPP_1, low; MPP_2, moderate; MPP_3, high).

## 3. Results

### 3.1. Instrumental Analysis

Overall, five instrumental parameters—two for colour and three for texture—showed significantly different mean values in the two cheeses (Table 4). Cheese produced with pasture milk and a longer ripening period was more yellow, having a higher b* index, whereas cheese produced with stall milk was lighter, having a higher L* index. Three out of nine texture parameters showed significantly different mean values between the two cheeses. Cheese produced with pasture milk and a longer ripening period was harder, resistant, and more elastic, showing greater values for linear distance force (computation of the force curve length), delta force (difference between yield force and final force), and elasticity modulus, computed as the ratio between stress and strain.

### 3.2. Consumer Panel Profile

A total of 156 subjects (55% men) aged between 18 and 75 took part in the test (Table 5). From the analysis of socio-demographic data, it was found that the participants had a high level of education: 50% declared a secondary school and 31% a bachelor’s/master’s degree. Furthermore, 73% reported to live with their families, mainly in urban areas (60%), and 48% did not have children. With regard to lifestyle and behavioural habits, it emerged that consumers adopted an average healthy lifestyle: 63% claimed they had never smoked and 53% practiced sports up to twice a week.

Regarding eating habits, the majority of the participants (69%) were omnivorous, 28% flexitarian (60% of them mainly lead a diet in which the consumption of meat was limited), and 3% vegetarian. As the tested product was of animal origin, vegans did not participate in the study.

With regard to attention to organic food, only 10% of the participants stated that organic products comprised more than 40% of their weekly shopping. Consumers, on the other hand, were attentive to the purchase of zero food miles products and to limiting food waste: The majority (76%) declared throwing away less than 5% of their weekly shopping.

A total of 44% of the consumer panel quite often organized excursions to the mountain huts (malga), mainly in Trentino province (87%). On these occasions, among the dairy products locally produced, they chose to buy fresh (33%) and mature cheese (33%), butter (16%), yogurt (10%), and milk (4%). The list of statements on mountain pasture practices and relative average scores and standard deviations are shown in Table 3. All statements obtained a high average score, demonstrating how the consumers associated positive opinions with mountain pasture practices. The only exception was the second statement (“Both stable and pasture management have the same impact on climate change”), which divided the opinion of the participants. However, only 33% of participants were aware of the possibility of buying cheeses with the “Sapori di Montagna” label directly from the supermarket.

### 3.3. Consumer Segmentation

Before classifying the consumers, according to the scales, the internal validity of each scale was verified (Table 6). All scales were reliable, showing standardized Cronbach’s alfas higher than 0.6 [59]. The NPI scale revealed a lower, but still comparable, internal validity, in comparison with that originally described by Roininen et al. [49] (Cronbach’s alpha = 0.76). In Table 6, the percentages of people in the three groups (low, moderate, and high interest/attitude), calculated for each scale according to the 33rd and 66th percentiles, are reported.

We found some associations with gender and age: Women (*p* = 0.033) and older respondents (*p* = 0.035) rated FCS items higher than men or younger respondents, respectively. Similar results for older consumers were obtained in the NPI and MPP knowledge scales (*p* < 0.05).

Additionally, those who showed a higher NPI declared a higher weekly purchase of organic and zero food miles products (*p* = 0.0004 and *p* = 0.006, respectively), and declared to be mainly flexitarians and vegetarians (*p* = 0.002). Participants who showed a higher attitude towards FCS declared less than 5% of weekly food waste (*p* = 0.003), more than 40% of weekly purchase of zero food mile products (*p* = 0.006), and limited or no meat consumption (*p* = 0.09).

**Figure 2:**
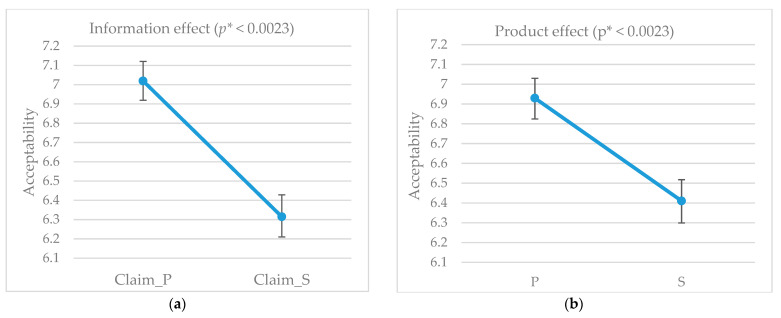
Effects of main factors in the conjoint study for the whole panel: (**a**) Information effect; and (**b**) Product effect. Bonferroni corrected p* is reported.

In the first row of Table 7, the results of the ANOVA mixed model for the whole consumer panel are reported. The main factors of product and external information had significant effects on consumer liking scores. Among the interaction effects, only that between consumer and product (C × P) was significant; showing that, among all consumers, people with different liking for products were present. External information had the strongest effect (MS = 76.16), with products claimed as “mountain pasture product” (Claim_P; M = 7.0, SD = 1.4) being statistically more preferred than those claimed as “valley floor stall product” (Claim_S; M = 6.3, SD = 1.6), regardless of the cheese effectively tasted (Figure 2a). Product effect was the second strongest (MS = 42.06), with more seasoned and pasture cheese (P; M = 6.9, SD = 1.5) being statistically different and more preferred than the stall one (L; M = 6.4, SD = 1.6); see Figure 2b.

The results of the ANOVA mixed model, recalculated for specific sub-groups of consumers identified by gender, age, altitude and urbanization of their place of residence, ATS, FCS, NPI, and opinion about MPP are reported in Table 7. Findings concerning the sub-groups of men, of people between 31 and 50 years old, of those who live in mountain areas, of those who had low ATS, high NPI, and positive opinions on MPP confirmed those observed for the overall panel of consumers (first row of Table 7). The random main consumer effect was not significant for any of the sub-groups, except for the group of people with high FCS attitude.

The most interesting results concerned the main effects of product and external information. The effect of the information was significant for all consumer groups: Men and women, people with different ATS, and so on, always evaluated the cheeses presented with the claim “mountain pasture product” with higher liking scores. The only exception lay in those who had a low opinion of MPP, who were not influenced by this information. The product effect was significant for a few sub-groups of consumers, in which mountain pasture cheeses showed higher liking scores, in comparison with valley floor stall cheeses. Mountain pasture cheese was more appreciated in the group of men, people aged between 31 and 50 years old, and those who had a very positive opinion on mountain pasture practices, although the effect of information remained the most important (Table 7, in parenthesis). Furthermore, mountain cheese was also more appreciated by those who lived above 600 m a.s.l. than those at lower altitude, as well as by those who had higher FCS attitude and NPI than those who were less attentive to these food aspects: In these groups, the product effect was a more important factor than information (Table 7, in parentheses). Furthermore, living in a more urban area or having a different attitude towards the social, economic, and environmental aspects of sustainability did not influence the significant effect of information on mountain production. Surprisingly, in the group of those who were less attentive to sustainability aspects, the product effect was the most important factor, showing a significant preference for mountain pasture cheese. Furthermore, there were changes in the significance of interaction effects between consumer and design factors on liking, which varied depending on the sub-group. In any case, the significant presence of these effects meant that, even within consumer sub-groups, different opinions were possible, both in the evaluation of the product and of the information.

## 4. Discussion

### 4.1. Mountain Cheese Acceptability

In the present paper, the acceptability of typical local cheeses produced in the same dairy from either mountain pasture or valley floor stall milk was investigated. Overall, both cheeses achieved good consumer acceptability, obtaining average liking scores ranging from 6.0 to 7.3. These results confirm what previous studies have stated, even without a real product tasting, as the quality of dairy products is an important aspect for both tourists and local farmers in the perception of summer mountain farms [3,20]. The significant effect of the product found for the whole panel (first row of Table 7) demonstrated that pasture cheese was significantly preferred over the stall one. This is in line with the results of Romanzin et al. [28], who reported that consumers expressed a higher actual liking for mountain pasture Montasio cheese. However, the cheeses evaluated here had different ripening and were different (as demonstrated by instrumental analyses), both in terms of colour and texture, with the pasture cheeses being harder and more yellow. Colour and texture generally change with the aging of dairy products [62,63], even if the yellow colour in milk and cheese also highly depends on their carotenoid content, which is generally higher in spontaneous pasture [64]. Our finding confirms that mountain pasture dairy products are recognizable and distinguishable from valley floor stall products [17,18]. Nevertheless, product sensory differences due to different milk production were probably influenced by differences induced by maturation [65], as the pasture cheeses were ripened 100 days more than the stall ones.

### 4.2. Information Effect

For the whole consumer panel, cheese acceptability was influenced by external information about the milk used for cheese manufacturing, showing that the “mountain pasture product” claim generated higher liking scores. This effect remained true for all the consumer segments investigated, except for people with a less positive opinion of mountain pasture practices (MPP1), who seemed not to be influenced by this information. Moreover, there were no effects due to gender, age, different area of residence, or different level of interest to natural products or different awareness of sustainable aspects (i.e., neither environmental nor food).

The strength and persistence of the external information effect on acceptability in the various groups of consumers surprised the authors, who expected a non-significance for the overall sample and significant effects in the groups of consumers who were more aware or sensitive to the information given [32,66]. This, in itself, is an important result; perhaps being due to the more detailed way in which the information was transmitted (i.e., a short sentence on the origin of the milk, accompanied by an image of the animal grazing or in the stable, depending on the level of information; Figure 1). Osburg et al. [67] reported, in fact, that more detailed information on the product increases both the purchase intention and the trust in eco-friendly products.

It is also possible that the image itself, in addition to reinforcing the concept already expressed in the claim, carries other concepts connected to the main information, such as animal welfare, naturalness, and sustainability issues that, when connected to food, also indicates a local, organic, and traditional product [68]. It is also known that visual imagery is an effective way to inform consumers and capture their interest, but also to increase the perceived product benefits [69]. Furthermore, the image linked to the mountain pasture cheese message used in this study was predominated by the colour green, which has been demonstrated to be associated with environmental friendliness and is the most effective for producing positive attitudes [70]. Previous studies that have examined consumer perception on verbal versus pictorial claims reached opposite conclusions on which modality is most effective [71,72]. Hence, further studies in this sense are necessary, in order to deepen the understanding of the message actually perceived by the consumer and to establish the most effective modality, passing from the evoked concept to a claim on packaging.

### 4.3. Segmentation Effect

The segmentation scales used in the present work—both those developed for this study and those developed by other authors—proved to be sufficiently reliable and with good external interpretability. The FCS scale, developed to measure attitudes towards sustainable food-related behaviours, was effective in assigning higher scores to people producing less domestic waste, purchasing a greater percentage of zero food miles and organic products, and people following a diet that limits or refuses meat consumption. The MPP scale, developed to investigate consumer opinions and knowledge about mountain pasture practices, was efficient in identifying consumers with different sensitiveness to external information about mountain pasture cheese.

Gender and age effects were found for the FCS, MPP, and NPI scales: Women and older consumers had higher scores with respect to food sustainability, mountain pasture practices aspects, and natural food interest. This finding was in line with our expectations: Women and older consumers are generally more attentive to the ecological aspects of food [36,73,74,75]. Cavaliere and Ventura [68], instead, argued that millennials are the most sustainable and environmentally friendly generation, even if their study did not include comparisons with groups of respondents aged over 30 years. The gender effect is also controversial. Muratore and Zarba [76] found that environmental aspects are more important to males, whereas other studies did not find any gender influence [77]. In our case, both genders were sensitive to information given; the difference was perhaps due to the different evaluative cues used by the two groups. Rahman et al. [78] saw that, in the evaluation of the sustainable aspects related to the production of garments, females were more sensitive to aspects of animal welfare, while males were more attentive to environmental aspects, such as air quality or the quantity of water used in production.

For mountain-related factors, those who lived in mountain areas (more than 600 m a.s.l) showed significant effects for both information and product factors; the latter was also the most important factor in this group of people. This may be due to the fact that inhabitants of mountain areas, compared to those who live in the plains or hills, are more familiar with the mountain pasture products, recognizing their sensory characteristics and associating them as local and zero food miles products.

The segmentation between those who lived in urban and rural areas did not lead to differences: There was no significant preference for one cheese over the other, while information on the mountain pasture product had a significant effect on liking in both groups. These results are contrary to those found by Zuliani et al. [2], who revealed a gap between urban consumer conception of mountain farming and the actual farming practices. The authors would have expected a non-significant effect of information in this group of consumers, with less knowledge of farming practices. This aspect is in line with the results obtained by segmenting consumers on the basis of their opinions on mountain pasture practices: Those with a less positive opinion were not influenced by external information, while those with a moderately positive opinion were. Those who had a highly positive opinion of MPP were not only sensitive to information given but also recognized the mountain pasture product as a better product, even if the information factor remained the most important one.

It is well-known that consumers are willing to pay a premium price to support local and organic food [24,79,80,81,82,83]. In our findings, segmentations based on FCS and NPI—which investigate attitudes towards organic and local products—did not show any difference in terms of external information influence. Instead, there was a difference in terms of product influence: Those with a high FCS and NP attitude actually preferred mountain pasture cheese and, thus, were more attentive to the aspects related to the product, which was also the most important factor.

Consumer segments with different attitudes towards socio-economic and environmental sustainability showed the same sensitivity to information. People presenting moderate or high attitudes seemed less attentive to the products: Those less engaged with sustainability were those who preferred mountain pasture cheese over the stall one. These results can be partly explained by the fact that, although concern and awareness of environmental problems are decisive for individual choices, the correlation between these aspects and actual behaviour is weak [84]. Furthermore, possessing environmental values, being aware of environmental problems, and having a correct perception of one’s own ecological footprint are necessary, but not sufficient, conditions to generate pro-environmental behaviour [19,36,85,86]; and, perhaps, not even to recognize these positive externalities in a food product.

### 4.4. Limitations and Future Perspectives

Our sample size of 156 subjects can be seen as a limitation. However, in the field of sensory sciences, the size of the consumer sample for an acceptability test is commonly recognised as adequate at around 100 consumers [87], even though the sample size from previously published research in the field commonly exceeds 100–120 subjects [88]. Thus, a consumer panel of 156 consumers can be considered an acceptable sample size, also because participants shared a core common feature (i.e., being potential consumers of dairy products), which represents the target demographic under investigation. In addition, our sample was balanced for gender and age classes.

All participants attended the cheese fair “La Casolara”, which is both an advantage and a limitation for the purposes of this study. If, on one hand, it is possible to reach a large number of cheese consumers in a short time; on the other hand, despite the ability of these events to attract not only local visitors, the majority of the consumers came from the same region. The external validity of our findings would surely be enhanced by considering a more representative sample of consumers coming from various regions. Furthermore, the preference for local food products—and, thus, mountain pasture products—could be related to the regional ethnocentrism of consumers [89], due to the close connection between local traditional practices and regional origin. We tried to measure this connection by classifying the subjects by area of origin, in terms of altitude and degree of urbanization, assuming a closer link for those who lived in a rural or mountain area.

Some of the variables included in this study were directly or indirectly measured using scales, while others were self-reported. It should be noted that the self-reported variables could be inaccurate, due to memory recall and subject bias [90]. Thus, in order to avoid the distortion of responses to different test instruction presentations, all the instructions were not given verbally but submitted on a screen, being the test anonymously administrated on a computer.

The study could be replicated by increasing the sample size and screening for regular cheese consumption. Future research could generalize the results obtained here into a national context, in order to identify the drivers promoting the introduction of these products into new markets. It would be interesting to repeat the test using other, more well-known types of cheese, such as parmesan, or some other types for which the sign of quality of “mountain product” is sought. Furthermore, the liking of other mountain products, such as butter or yogurt, which are sold directly in the mountain hut could be investigated. Finally, future research could also be dedicated to the study of the images and claims used to evoke the concept of valley floor stall and mountain pasture products, identifying the most effective ones in a large validation study.

## 5. Conclusions

The present study showed that the impact of “mountain product” information on the acceptability of local cheese generated an overall positive response in the consumers, who assigned it extra value. The importance of this extrinsic characteristic exceeded the intrinsic value of the tasted product, which, however, was globally recognized, even if to a lesser extent. The positive effect of information persisted even within groups of consumers with different socio-environmental characteristics and different levels of interest and attitude. This study also showed that the consumers generally associated positive opinions with mountain pasture practices; it was precisely the lack of this positive association that made the claim lose its effectiveness. Furthermore, the consumers who lived in mountainous areas, who had a high opinion of mountain pasture practice, and who were predisposed towards local and organic food products and sustainable food-related behaviours were able to identify mountain pasture cheese as a product of higher quality than the valley floor stall cheese. This study contributes to revealing that the foundations exist for mountain pasture products to become mainstream products for all consumers. Nevertheless, effort is needed to promote the product in places other than mountain huts or dairy vendors.

## Figures and Tables

**Table 1:** Demographics, food behaviour questions, attitude questionnaires, and their relative acronyms, number of items, rating scale, response options, and references.

Topic	Questionnaire/ Question	Items	Scale and Response Options	References
Demographic	Age	4	Completed years (open answer)	Developed by the authors
	Educational qualification		None, Primary, Lower secondary, Upper secondary, Bachelor/Master degree, Post-graduate degree	
	Family		Alone, In family, Other	
	Number of children		From 0 to 3 or more	
Food behaviour and life style	Smoking habit	6	Never tried, gave up, occasionally, regularly	Developed by the authors
	Sport		No, Up to twice a week, More than twice a week	
	Food Diet		Omnivores, flexitarians, vegetarians and vegans; classification based on the eating diet chosen out of a list of ten	Adapted from De Backer & Hudders [46]
	Organic food weekly purchased		<5%, 5–10%, 11–20%, 21–30%, 31–40%, >40%	Developed by the authors
	Zero food miles food weekly purchased		<5%, 5–10%, 11–20%, 21–30%, 31–40%, >40%	
	Food waste weekly throw it away		<5%, 5–10%, 11–20%, 21–30%, 31–40%, >40%	
	HTAS *—Natural product Interest domain (NPI)	6	9-point Likert scale (1 = totally disagree; 9 = totally agree)	Roininen et al. [49] (Table S1)
Sustainability	Attitude Towards Sustainability (ATS)	15	9-point Likert scale (1 = totally disagree/not at important/not at all concerned; 9 = totally agree/very important/very concerned, depending on the item)	Poortinga & Darnton [39] (Table S2)
	Food Consumption Sustainability (FCS)	18	9-point Likert scale (1 = totally disagree; 9 = totally agree)	Developed by the authors (Table 2 and Table S3)
Mountain	Area of residence	7	Urban (>150 inhabitants/km^2^), Rural (<150 inhabitants/km^2^)	Developed by the authors
	Altitude of residence		>600, 300–600, <300 m a.s.l.	
	Mountain hiking		Never, rarely, often, always	
	Hiking zone		Trentino, Alto-Adige, out of region	
	Mountain pasture (MP) product purchasing		Never, rarely, often, always	
	MP products purchased		Fresh cheese, mature cheese, butter, yogurt, milk, more	
	MP cheese sold at supermarket (knowledge)		Yes, No	
	MP Practice Values (MPP)	6	9-point Likert scale (1 = totally disagree; 9 = totally agree)	Developed by the authors (Table 3 and Table S4)

* HTAS health and taste attitude scale.

**Table 2:** Mean (M) and Standard Deviation (SD) values for each statement of the Food Consumption Sustainability scale.

Item	Food Consumption Sustainability	M	SD
1	It is better to buy local foods because they cost less	6.24	2.30
2	It is better to buy foreign foods because they are cheaper ^R^	2.03	1.35
3	It is better to buy local food because it pollutes less	7.65	1.51
4	It is better to buy local food because local labour is employed	7.83	1.40
5	It is better to buy foreign food to have more choice ^R^	2.72	1.67
6	It is better to buy local foods because they are better	6.84	1.82
7	It is better to buy foreign foods because they are better ^R^	2.56	1.67
8	There are no advantages to buying local foods over foreign ones ^R^	2.40	1.79
9	I try to buy seasonal fruit and vegetables so I pollute less	7.46	1.85
10	It is better to buy seasonal fruit and vegetables because there is no need to transport them from afar	7.63	1.65
11	I buy the fruit and vegetables I want regardless of the season ^R^	3.65	2.25
12	In my opinion, eating only seasonal fruit and vegetables is unhealthy ^R^	1.93	1.53
13	I would be willing to pay more for environmentally friendly catering services	6.57	1.92
14	I would choose one food product over others if labelled as “green” ^R^	6.69	1.95
15	When I buy food, my priority is taste and value for money before “green” aspects	4.81	2.13
16	When I eat out, I would like to be offered local food and drink if possible	7.64	1.54
17	Rather than throwing away food, I eat it even if it is 1–2 days out of date	7.00	2.34
18	When I do the shopping, I always buy more than I need ^R^	3.66	2.06

^R^ Negative statements recoded for the final score calculation.

**Table 3:** Mean (M) and Standard Deviation (SD) values for each statement of the Mountain Pasture Practice scale.

Item	Statements about Mountain Pasture Practices	M	SD
1	The mountain pasture practice helps to maintain pleasant high mountain landscapes [52]	7.92	1.37
2	Both stable and pasture management have the same impact on climate change [53]	3.38	2.48
3	The mountain pasture practice contributes to the welfare of the animals [1,2]	8.12	1.17
4	The mountain pasture practice produces high quality dairy products [10,54]	8.04	1.14
5	The mountain pasture practice increases tourist activity [55]	7.85	1.39
6	The mountain pastures maintain a high natural animal and plant biodiversity [56,57]	7.89	1.35

**Table 4:** Instrumental characterisation of pasture (P) and stall (S) cheese: Means, standard deviations (in parenthesis), and *p*-values for colour and texture parameters.

Parameters	P	S	*p*-Value *
Lightness (L *)	71.9 (1.3)	75.6 (1.0)	0.001
Redness (a *)	−2.5 (0.2)	−2.4 (0.1)	0.116
Yellowness (b *)	25.8 (0.9)	17.8 (0.5)	0.001
Yield Force (F1)	3.8 (1.1)	3.0 (1.9)	0.199
Max Force (F2)	4.7 (0.6)	5.0 (0.6)	0.266
Final Force (F3)	4.6 (0.7)	4.9 (0.6)	0.234
Number of Force Peaks (FP)	1.2 (0.9)	0.8 (0.7)	0.965
Area (A)	350.3 (51.8)	321.0 (47.8)	0.259
Linear Distance Force (LDF)	91.0 (0.5)	90.6 (0.1)	0.001
Elasticity modulus (E)	0.4 (0.1)	0.2 (0.1)	0.001
Mean Force (F4)	4.1 (0.6)	4.1 (0.6)	7.259
Delta Force (DF)	−0.8 (1.1)	−1.9 (1,4)	0.011

* Bonferroni corrected *p*-values.

**Table 5:** Percentage distribution of socio-demographic characteristics by gender of respondents recruited in the consumer study.

	Males % (*n* = 85)	Females % (*n* = 71)	Total (*n* = 156)
*Age (years)*			
18–30	24.7	29.6	26.9
31–50	47.1	43.7	45.5
50–75	28.2	26.8	27.6
*Educational level*			
None	0.0	1.4	0.6
Primary	2.4	1.4	1.9
Lower secondary	10.5	7.0	9.0
Upper secondary	49.4	50.7	50.0
Bachelor/Master degree	31.8	29.6	30.8
Post-graduate	5.9	9.9	7.7
*Who do you live with?*			
Alone	14.1	18.3	16.0
In family	77.6	67.6	73.1
Other	8.2	14.1	10.9
*N° of children*			
None	47.1	47.9	47.4
One	16.5	14.1	15.4
Two	29.4	32.4	30.8
Three or more	7.1	5.6	6.4
*Area of residence*			
Urban (>150 inhabitants/km^2^)	61.2	57.7	59.6
Rural (<150 inhabitants/km^2^)	38.8	42.3	40.4
*Altitude of residence*			
>600 m a.s.l.	25.9	22.5	24.4
300–600 m a.s.l.	30.6	29.6	30.1
<300 m a.s.l.	43.5	47.9	45.5
*Smoking*			
Not smoking (never tried)	57.6	69.0	62.8
Not smoking (quit)	31.8	8.5	21.1
Occasionally	3.5	15.5	9.0
Regularly	7.1	7.0	7.1
*Sport*			
No	10.6	22.5	16.0
Up to 2 times a week	55.3	50.7	53.2
More than 2 times	34.1	26.8	30.8
*Diet*			
Omnivores	77.6	59.1	69.2
Flexitarians	22.4	33.8	27.6
Vegetarians	0.0	7.0	3.2

**Table 6:** Means (M), standard deviations (SD), percentile cut-points (33rd and 66th), percentage of participants in each group (L = low attitude; M = moderate attitude; H = high attitude), and reliability of attitude scales: Natural product interest (NPI), attitudes toward sustainability in general (ATS), food consumption sustainability (FCS), and mountain pasture practice scale (MPP).

Scale	M	SD	33rd	66th	L (%)	M (%)	H (%)	Cronbach’s α	Standardized α
NPI	37.3	8.4	33	41	29	34	37	0.66	0.66
ATS	91.8	11.0	88	96	30	38	32	0.57	0.64
FCS	124.0	13.3	118	131	31	34	35	0.69	0.73
MPP	43.2	5.8	42	46	30	28	42	0.68	0.79

**Table 7:** The *p*-values of ANOVA mixed model on the effects of consumer, conjoint factors and their second order interactions (*) on liking. Mean squares are reported in parentheses. Statistically significant effects after Bonferroni correction (*p*-value < 0.0022) are reported in bold.

Groups	N	Consumer (C)	Product (P)	Information (I)	P*C	I*C	P*I
All	156	0.014 (4.18)	**0.0001 (42.1)**	**0.0001 (76.2)**	**0.0001 (2.6)**	0.059 (1.3)	0.467 (0.5)
M	85	0.019 (3.8)	0.001 (30.6)	**0.0001 (36.9)**	**0.0001 (2.4)**	0.815 (0.9)	0.838 (0.1)
F	71	0.163 (4.7)	0.039 (12.7)	**0.0001 (39.6)**	**0.0001 (2.6)**	**0.001 (1.7)**	0.36 (0.7)
Age_1	42	0.409 (3.9)	0.042 (13.7)	**0.0001 (25.9)**	**0.0001 (3.1)**	0.013 (1.0)	0.057 (1.9)
Age_2	71	0.056 (4.7)	**0.001 (33.3)**	**0.0001 (35.9)**	**0.001 (2.7)**	0.078 (1.7)	0.628 (0.3)
Age_3	43	0.039 (3.5)	0.362 (1.7)	**0.0001 (15.1)**	0.022 (2.0)	0.812 (0.8)	0.508 (0.5)
Alt_1	38	0.464 (3.5)	**0.002 (33.2)**	**0.0001 (19.9)**	**0.0001 (3.0)**	0.11 (1.2)	0.066 (2.9)
Alt_2	47	0.005 (5.2)	0.007 (13.3)	**0.0001 (24.6)**	0.021 (1.7)	0.029 (1.6)	0.543 (0.3)
Alt_3	71	0.16 (5.9)	0.163 (5.9)	**0.0001 (31.8)**	**0.0001 (3.0)**	0.443 (1.1)	0.078 (3.4)
Urb_1	93	0.027 (3.7)	0.005 (20.8)	**0.0001 (42.7)**	**0.0001 (2.6)**	0.09 (1.3)	0.247 (1.3)
Urb_2	63	0.137 (4.5)	0.006 (21.7)	**0.0001 (33.6)**	**0.0001 (2.7)**	0.193 (1.3)	0.803 (0.1)
NPI_1	45	0.104 (4.3)	0.078 (8.9)	**0.0001 (20.0)**	0.012 (2.7)	0.922 (1.9)	1 (0.0)
NPI_2	53	0.09 (4.3)	0.378 (1.9)	**0.0001 (31.7)**	**0.0001 (2.4)**	**0.002 (1.4)**	0.118 (1.5)
NPI_3	58	0.103 (3.6)	**0.0001 (44.8)**	**0.0001 (24.9)**	**0.0001 (2.6)**	0.1 (1.5)	1 (0.0)
ATS_1	47	0.038 (4.4)	**0.0001 (42.3)**	**0.0001 (29.9)**	0.024 (1.9)	0.078 (1.6)	0.234 (1.5)
ATS_2	59	0.215 (4.3)	0.89 (9.8)	**0.0001 (23.2)**	**0.0001 (3.3)**	0.242 (1.0)	0.887 (0.1)
ATS_3	50	0.127 (3.6)	0.252 (3.1)	**0.0001 (23.8)**	0.004 (2.3)	0.308 (1.3)	0.84 (0.1)
FCS_1	49	0.569 (2.3)	0.651 (0.1)	**0.0001 (12.3)**	0.003 (3.0)	0.972 (0.8)	0.951 (0.1)
FCS_2	53	0.157 (4.2)	0.043 (10.4)	**0.0001 (37.4)**	**0.0001 (2.4)**	0.013 (1.7)	0.513 (0.4)
FCS_3	54	**0.002 (6.0)**	**0.0001 (50.1)**	**0.0001 (29.6)**	**0.0001 (2.2)**	0.032 (1.3)	0.446 (0.5)
MPP_1	48	0.552 (4.0)	0.092 (11.0)	0.003(14.1)	**0.0001 (3.7)**	0.124 (1.4)	0.387 (0.8)
MPP_2	43	0.019 (4.2)	0.115 (5.2)	**0.0001 (15.7)**	0.004 (2.0)	0.47 (0.9)	0.418 (0.6)
MPP_3	65	0.022 (4.0)	**0.001 (28.5)**	**0.0001 (50.0)**	**0.001 (2.2)**	0.123 (1.4)	0.228 (1.5)

## Data Availability

The data generated during and/or analyzed during the current study are available from the corresponding author on reasonable request.

## Supplementary Materials

The following are available online at https://www.mdpi.com/2304-8158/10/3/682/s1. Table S1: List of the original statements of the Natural Product Interest domain of the Heath and Taste Attitude Scale (HTAS) (a) and the translation in Italian (b). Table S2: List of the original statements of the Welsh Sustainability Segmentation Screening Tool (a) and its adaptation in Italian (b). Table S3: List of the statements of the Food Consumption Sustainability scale in Italian. Table S4: List of the statements of the Mountain Pasture Practice scale in Italian.
