# Supplementary material for: Does the ‘Mountain Pasture Product’ Claim Affect Local Cheese Acceptability?
Source: Foods. 2021 Mar 23;10(3):682. doi: 10.3390/foods10030682 (PMC8005200; doi:10.3390/foods10030682)
Supplement: Supplementary file 1 [file foods-10-00682-s001.pdf]

foods MDPI

Supplementary materials

Table S1. List of the original statements of the Natural Product Interest domain of the Heath and Taste Attitude Scale (HTAS) (a) and the translation in Italian (b).

**(a) HTAS - Natural Product Interest domain (Roininen et al., 1999)**	
1	I try to eat foods that do not contain additives
2	I do not care about additives in my daily diet [R]
3	I do not eat processed foods, because I do not know what they contain
4	I would like to eat only organically grown vegetables
5	In my opinion, artificially flavoured foods are not harmful for my health [R]
6	In my opinion, organically grown foods are no better for my health than those grown conventionally [R]
**(b) HTAS - Natural Product Interest domain (Italian version)**	
1	Cerco di mangiare cibi che non contengono additivi
2	Nella mia dieta quotidiana non presto attenzione agli additivi [R]
3	Non mangio cibi trasformati perché non so cosa contengono
4	Vorrei mangiare solo verdura biologica
5	Secondo me, i prodotti che contengono aromi artificiali non sono nocivi per la salute [R]
6	Per me, i cibi biologici non sono più sani di quelli coltivati con metodo tradizionale [R]

[R] Negative statements recoded for the final score calculation.

Table S2. List of the original statements of the Welsh Sustainability Segmentation Screening Tool (a) and its adaptation in Italian (b). Response options varying according to the statement from item 1 to 4 (1 = not important at all; 9 = extremely important), for item 12 (1 = not concerned at all; 9 = extremely concerned), for all other item (1=totally disagree; 9 = totally agree).

Item	Welsh Sustainability Segmentation Screening Tool (Poortinga, W. & Darnton, A., 2016)	Italian Attitude Towards Sustainability (ATS)
**1**	Protecting the environment (preserving nature)	Proteggere l'ambiente (preservare la natura)
**2**	Being influential (having an impact on people and events)	Essere influenti (avere un impatto su persone ed eventi)
**3**	Sense of belonging (feeling that others care about me)	Senso di appartenenza (sentire che gli altri si prendono cura di te)
**4**	Being independent (self-reliant, self-sufficient)	Essere indipendenti (autonomi, autosufficienti)
**5**	If things continue on their current course, we will soon experience a major environmental disaster	Se le cose non cambieranno, si verificherà presto un grave disastro ambientale
**6**	People who fly should bear the cost of the environmental damage that air travel causes	Le persone che viaggiano in aereo dovrebbero pagare il costo del danno ambientale causato dal viaggio
**7**	It is very important for Wales to have a high level of economic growth	È molto importante per l'Italia avere un alto tasso di crescita economica
**8**	There are much more important things for me to do than protect the environment	Ci sono cose molto più importanti da fare che tutelare l'ambiente [R]

**9**	Economic growth and creating jobs should be the top priority, even if environment suffers	La crescita economica e la creazione di posti di lavoro dovrebbero essere le nostre priorità assolute, anche se l'ambiente ne risentirebbe [R]
**10**	Being green is an alternative lifestyle: it's not for the majority	Essere ambientalisti è uno stile di vita alternativo: non è per la maggioranza [R]
**11**	The effects of climate change are too far in the future to really worry me	Gli effetti del cambiamento climatico sono troppo lontani nel futuro perché io me ne preoccupi davvero [R]
**12**	Supplies of fossil fuels (e.g. coal and gas) will run out: how concerned are you about the future?	Le scorte di combustibili fossili (ad es. carbone e gas) si esauriranno: quanto ti preoccupa il futuro?
**13**	I feel like I belong to this neighbourhood	Mi sento di appartenere al mio quartiere/alla mia comunità
**14**	If I were to move I would like to live in a similar place to where I live now	Se dovessi trasferirmi mi piacerebbe vivere in un posto simile a quello in cui vivo ora
**15**	We should act to protect the Welsh landscape so it can be enjoyed by future generations	Dovremmo agire per proteggere il paesaggio italiano in modo che ne possano godere le generazioni future

[R] Negative statements recoded for the final score calculation.

Table S3. List of the statements of the Food Consumption Sustainability scale in Italian.

Item	Food Consumption Sustainability Scale (Italian version)
1	E' meglio comprare alimenti locali perché costano meno
2	E' meglio comprare alimenti esteri perché costano meno [R]
3	E' meglio comprare alimenti locali perché così si inquina meno
4	E' meglio comprare alimenti locali perché si dà lavoro alla manodopera locale
5	E' meglio comprare alimenti esteri per avere più scelta [R]
6	E' meglio comprare alimenti locali perché sono migliori
7	E' meglio comprare alimenti esteri perché sono migliori [R]
8	Non ci sono vantaggi a comprare alimenti locali rispetto ad alimenti esteri [R]
9	Cerco di comperare frutta e verdura di stagione così inquino meno
10	E' meglio comperare frutta e verdura di stagione perché non c'è bisogno di trasportarla da lontano
11	Compero frutta e verdura che voglio indipendentemente dalla stagione [R]
12	Secondo me mangiare solo frutta e verdura di stagione è poco salutare [R]
13	Sarei disposto a pagare di più per servizi di ristorazione rispettosi dell'ambiente
14	Sceglierei un prodotto alimentare rispetto ad altri se fosse etichettato "verde" [R]
15	Quando compero degli alimenti la mia priorità sono il gusto e il rapporto qualità-prezzo prima di aspetti "verdi"
16	Quando mangio fuori casa vorrei che mi fossero offerti cibo e bevande di provenienza locale se possibile
17	Piuttosto che buttare del cibo lo mangio anche se è scaduto da 1-2 giorni
18	Quando faccio la spesa compero sempre più del necessario [R]

[R] Negative statements recoded for the final score calculation.

Table S4. List of the statements of the Mountain Pasture Practice scale in Italian.

Item	Mountain Pasture Practice scale (Italian version)
**1**	La pratica dell'alpeggio contribuisce a mantenere gradevoli paesaggi di alta montagna
**2**	Sia la gestione in stalla che in alpeggio hanno lo stesso impatto sul cambiamento climatico
**3**	La pratica dell'alpeggio contribuisce al benessere degli animali
**4**	La pratica dell'alpeggio produce prodotti lattiero-caseari di alta qualità
**5**	La pratica dell'alpeggio accresce l'attività turistica
**6**	L'alpeggio mantiene un'alta biodiversità naturale animale e vegetale
